# Case Report: From complete remission to severe anemia: pure red cell aplasia emerged after VRd regimen in multiple myeloma

**DOI:** 10.3389/fonc.2026.1904201

**Published:** 2026-08-19

**Authors:** Xiaoqing Zhang, Ting Hu, Hongmei Dai, Hua Yuan

**Affiliations:** Department of Nephrology and Hematology, Yunyang County People’s Hospital, Chongqing, China

**Keywords:** anemia, immune dysfunction, lenalidomide, multiple myeloma, PrCa

## Abstract

Pure red cell aplasia (PRCA) is a rare disorder characterized by severe reticulocytopenia and absence of erythroid precursors. Its association with multiple myeloma (MM) therapy is uncommon. We report a 73-year-old man with MM (IgGλ, stage IIIA) who achieved complete remission after five cycles of VRd (bortezomib, lenalidomide, dexamethasone). During lenalidomide maintenance, he developed severe anemia (hemoglobin 45 g/L, reticulocytes 0.01%). Bone marrow biopsy showed absent erythroid precursors with preserved granulocytic and megakaryocytic lineages, consistent with PRCA. Following discontinuation of lenalidomide, cyclosporine therapy increased hemoglobin to 92 g/L within one month. This case highlights that PRCA can occur after MM treatment and underscores the importance of early detection by monitoring reticulocytes. A comprehensive approach including withdrawal of offending agents and immunosuppressive therapy is critical.

## 1 Background

PRCA is a rare disorder defined by normocytic anemia, severe reticulocytopenia, and the absence or near-absence of erythroid precursors in the bone marrow (1). Acquired PRCA, the more common form, can be idiopathic (primary) or secondary to a range of factors, including viral infections (e.g., HCV, HIV, EBV, parvovirus B19), autoimmune conditions, drugs, and neoplasms (1).

In recent years, the link between PRCA and plasma cell dyscrasias has received growing attention. In a cohort of 51 patients with PRCA, 24% had an underlying monoclonal gammopathy, monoclonal gammopathy of undetermined significance (MGUS), or smoldering multiple myeloma (SMM); bone marrow findings included clonal plasma cell expansion, hypercellularity, and fibrosis (2, 3). It has been proposed that paraprotein may inhibit early erythropoiesis (2, 3). Of note, patients with PRCA and a coexisting monoclonal gammopathy who received anti-myeloma treatment showed reticulocyte recovery and became transfusion-independent, supporting a pathogenic contribution of plasma cells in this subset of PRCA (2).

Despite this association, the direct occurrence of PRCA in the setting of active MM is exceptionally rare. Qi Hu and colleagues recently described a patient with active MM who developed PRCA following lenalidomide-based therapy. Mechanistic work suggested that an abnormal interaction between activated CD8^+^ T cells and erythroid cells overexpressing MHC-class I might contribute to lenalidomide-induced PRCA in MM (4). Here we report a patient with MM who presented with PRCA after achieving complete remission (CR) on VRd treatment. A systematic workup excluded common secondary causes of PRCA. This case further underscores a possible link between MM therapy and the development of PRCA.

## 2 Case report

A 73-year-old man presented with abnormal serum globulin levels and was subsequently diagnosed with multiple myeloma [IgG λ type, Durie-Salmon stage IIIA, International Staging System (ISS) stage II, and revised ISS (RISS) stage II]. At diagnosis, his hemoglobin was 95 g/L, while white blood cell and platelet counts, as well as renal function, remained within normal limits. He received six cycles of VRd chemotherapy, achieving a best response of CR, defined by bone marrow plasma cells of 1%, negative serum M-protein by immunofixation, and a normal serum free light chain ratio. After completing the six cycles, lenalidomide maintenance therapy was started at 25 mg/day on a 21-day cycle.

**Figure 1:**
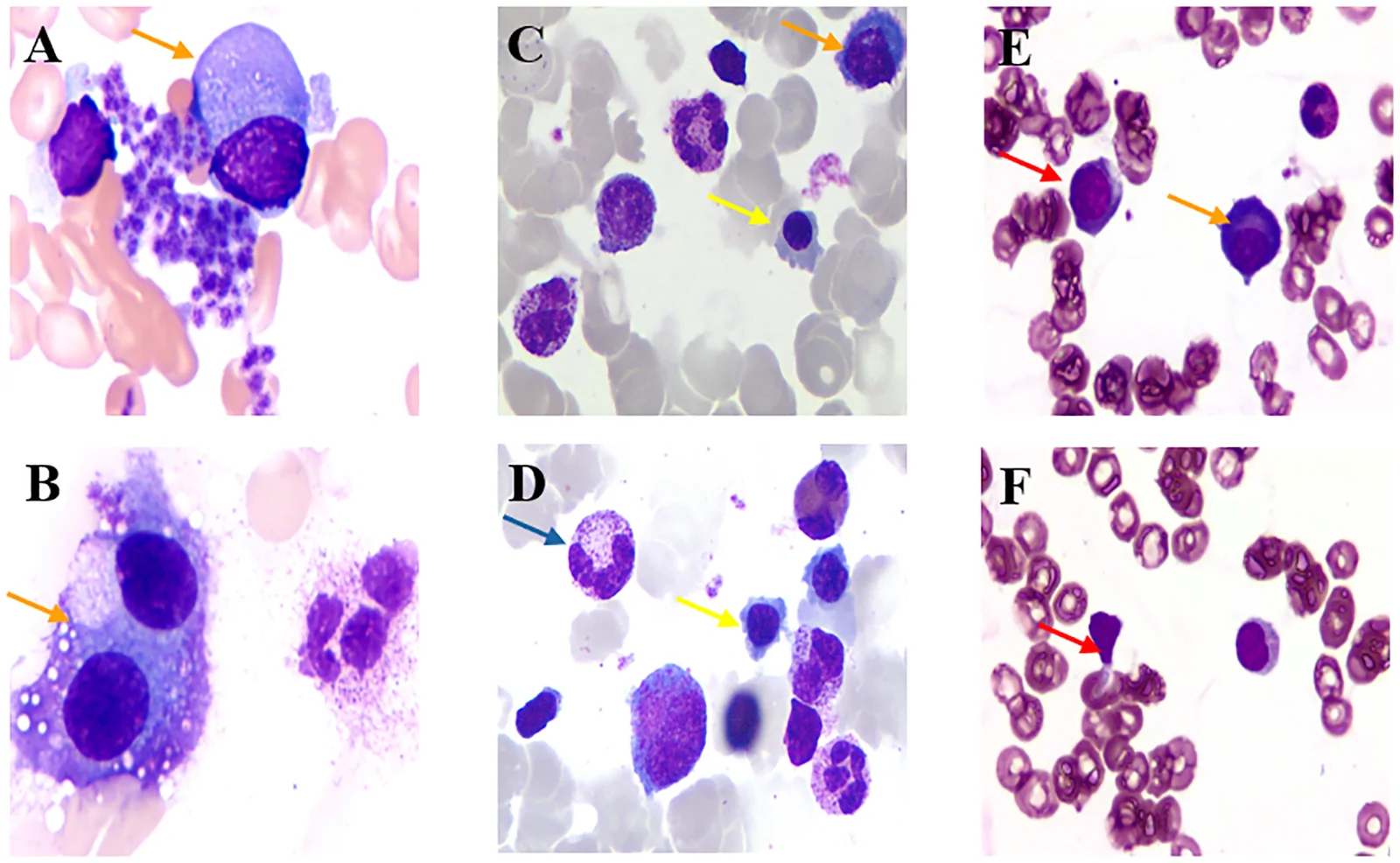
Bone marrow smears at different stages of the disease. **(A, B)** At initial diagnosis, marked infiltration by abnormal plasma cells is observed (orange arrows). **(C, D)** After VRd therapy, the marrow shows remission with normal plasma cells (orange arrows), and the cellularity is predominantly granulocytic (blue arrows) and erythroid (yellow arrows). **(E, F)** Following the development of pure red cell aplasia, the marrow exhibits marked erythroid hypoplasia, increased lymphocytes (red arrows), and occasional normal plasma cells (orange arrows).

During lenalidomide maintenance, however, his anemia progressively worsened: hemoglobin dropped to 45 g/L and the reticulocyte proportion fell to 0.01%. Serum vitamin B_12_, folate, and ferritin levels were all within normal limits. Testing for antinuclear antibodies, direct and indirect Coombs tests, and serological screens for HIV-1, HIV-2, hepatitis B surface antigen, Epstein–Barr virus, cytomegalovirus, and hepatitis C virus were all negative. Chest computed tomography showed no mediastinal mass. Bone marrow aspiration revealed active cellularity with decreased nucleated cell counts, erythroid hypoplasia (2% of nucleated cells), normal granulocytic and megakaryocytic morphology and proportions, and no aberrant plasma cells, thus ruling out other common causes of anemia. Bone marrow biopsy demonstrated an absence of erythroid precursors with preserved granulocytic and megakaryocytic lineages, and no abnormal myeloid or neoplastic cells were identified. These findings led to a diagnosis of PRCA (**Figure 1**; Bone marrow findings at different disease stages). Additionally, flow cytometry showed no abnormal CD55/CD59 expression, excluding paroxysmal nocturnal hemoglobinuria and other hematological disorders.

Given previous reports implicating lenalidomide as a potential trigger of PRCA, the drug was promptly discontinued. In accordance with PRCA treatment guidelines (5), cyclosporine was initiated at 150 mg once daily. Within two months, the patient’s hemoglobin rose from 45 g/L to 92 g/L (**Table 1**; serial bone marrow and anemia parameters at different stages).

**Table 1:** Serial bone marrow and anemia parameters at different stages.

Time	Apr 26, 2025	Jun 29, 2025	Sep 1, 2025	Nov 5, 2025	Feb 10, 2026	Mar 26, 2026
Item	Initial diagnosis	2*VRD	4*VRD	6*VRD,	Post-1-Month R maintenance, suspected PRCA	Post-2-Month R maintenance, PRCA diagnosis
M protein (g/L)	2.97	0	0	0	–	0
BMPC%	20.3	5	1	1	–	1
Beta-2 microglobulin (ug/L)	3.6	2.9	4.3	4.3	4.0	3.5
Hemoglobin (g/L)	96	106	82	82	65	45
RET%	2.10	1.22	0.51	0.51	–	0.01
ARC(×10^9^/L)	80.5	75.4	33,4	22.5	–	5.4
MCV(fL)	95	99	90	89	98	90
WBC(×10^9^/L)	8.8	5.6	4.5	3.9	8.9	6.7
PLT(×10^9^/L)	292	135	280	154	230	300

The abbreviation for the table, BMPC%, Bone marrow plasma cell percentage; RET%, Reticulocyte percentage; ARC, Absolute reticulocyte count; MCV, Mean corpuscular volume; WBC, White blood cell count; PLT, Platelet count.

## 3 Discussion

MM is a plasma cell neoplasm. Although anemia is a common end-organ manifestation of MM, PRCA with maturation arrest of erythroid precursors is not a typical finding (6, 7). The patient described here developed worsening anemia after achieving complete remission on VRd therapy. Normocytic normochromic anemia, severe reticulocytopenia (typically <1%), marked reduction of erythroid precursors (<5%) on bone marrow biopsy, and exclusion of other causes all pointed to PRCA.

In this patient with an established diagnosis of MM who developed PRCA during maintenance therapy, several etiologies warrant consideration, including plasma cell clone-related immune dysregulation, direct drug-induced effects, or a combination of both. Korde et al. observed that 24% of 51 patients with PRCA had an underlying monoclonal plasma cell disorder, and among those who received anti-myeloma treatment, reticulocyte recovery was noted, suggesting a functional link between plasma cells and erythroid precursors (2). The proposed mechanism involves paraprotein-mediated inhibition of early erythropoiesis (2, 3). However, in our patient, the achievement of CR after VRd induction, makes plasma cell-driven immune dysregulation less likely as the primary cause. Additionally, serological tests for Epstein–Barr virus, cytomegalovirus, and other common viruses were all negative, providing no evidence of occult infection. No CHIP-related mutations or overt immunosenescence markers were identified, further diminishing the likelihood of these alternative mechanisms.

Drug-induced PRCA deserves particular attention in the era of targeted and immunotherapies for multiple myeloma. Several case reports have described PRCA developing in patients receiving bortezomib, possibly through its broad immunomodulatory effects, though the exact mechanism remains unclear (8). Lenalidomide, a cornerstone of myeloma therapy, has been implicated in PRCA by both *in vitro* and clinical evidence (4, 9). Moreover, PRCA has been reported in patients with POEMS syndrome treated with lenalidomide; its discontinuation led to marked improvement of anemia, further supporting a causative role (9). T-cell abnormalities are thought to be central to drug-induced PRCA. In the study by Qi Hu et al., a patient developed PRCA after VRd therapy; single-cell sequencing and *in vitro* experiments revealed that lenalidomide facilitates the recognition and elimination of erythroid cells that aberrantly overexpress MHC class I by CD8^+^ T cells, highlighting T-cell immune dysregulation as a core mechanism—although this finding is based on a single case and requires validation in larger cohorts (4).

In our patient, it remains uncertain whether PRCA was secondary to lenalidomide or prior bortezomib exposure. However, several observations support a drug-related etiology: anemia improved during the VRd regimen (which included both agents), but worsened significantly during lenalidomide maintenance, and the bone marrow findings were consistent with PRCA (4, 9). Lenalidomide-induced PRCA has also been described in other hematological malignancies, such as myelodysplastic syndromes (10), suggesting that lenalidomide or the associated immune reconstitution may be a potential trigger, although a definitive causal relationship has not been established. Furthermore, anti-CD38 monoclonal antibodies have revolutionized myeloma treatment by targeting plasma cell surface CD38. Nevertheless, Saba et al. reported a case of PRCA with a DNMT3A mutation in a patient receiving daratumumab maintenance therapy (11). Anti-CD38 antibodies may deplete both clonal and non-clonal plasma cells that support erythropoiesis, and the ensuing immune reconstitution could promote aberrant T-cell activation, potentially facilitating PRCA development (12). Although our patient was not treated with daratumumab, this observation underscores the broader issue of immune-modulating agents as potential inducers of PRCA in the myeloma setting.

Taken together, the temporal association between the initiation of lenalidomide maintenance and the onset of severe anemia, combined with the rapid hematologic improvement after drug withdrawal and cyclosporine therapy, strongly supports an immune-mediated process most likely triggered by lenalidomide in this case. While other mechanisms cannot be entirely excluded, the cumulative evidence points to lenalidomide as the principal culprit.

From a clinical standpoint, when an MM patient develops unexplained severe anemia with pronounced reticulocytopenia, bone marrow aspiration and biopsy should be performed promptly to confirm or exclude PRCA. Individualized management strategies include withdrawing suspected offending drugs, optimizing anti-myeloma therapy, and initiating immunosuppressive regimens such as cyclosporine, corticosteroids, or antithymocyte globulin (3).

Nevertheless, several limitations of this case should be acknowledged. PRCA is a diagnosis of exclusion; in our patient, the diagnosis was based on peripheral blood counts and bone marrow morphology, but we did not perform parvovirus B19 testing or measure erythropoietin (EPO) levels. Furthermore, the follow-up period was relatively short, and as a single case report, this study lacks large-scale clinical validation. In addition, we were unable to obtain immunologic or mechanistic data that might further elucidate the underlying pathophysiology, which limits the generalizability of our findings.

In conclusion, PRCA occurring after treatment for MM is a rare and etiologically complex event that may involve both direct drug effects and plasma-cell-clone-associated T-cell immune dysregulation. A comprehensive strategy integrating anti-myeloma treatment with immune intervention is needed to improve anemia and maintain overall disease control. Despite these limitations, this case emphasizes the need for vigilant monitoring, prompt diagnosis, and further research into mechanisms and management of this rare complication.

## Data Availability

The raw data supporting the conclusions of this article will be made available by the authors, without undue reservation.
